# Treatment of Iritis

**Published:** 1883-01-20

**Authors:** A. G. Hobbs

**Affiliations:** Professor of Ophthalmology


### TREATMENT OF IRITIS.

By A. G. HOBBS, M.D.,

**Professor of Ophthalmology. Extract from a clinical lecture delivered at the Southern Medical College.**

The essential and master remedy in iritis is atropia. It is the alpha and omega—the beginning, the middle and the end.

The prevailing fault is to use it too sparingly. Its potency when the iris is inflamed is far less than when the eye is normal. The reasons are as follows: The power of endosmosis through the cornea is impaired because its tissues are surcharged with fluid and the tension of the globe is increased, and the swollen condition of the iris and the adhesions combine to oppose its effect even when the solution has entered the aqueous chamber. For these reasons, a solution of iv gr. to the oz. of atropia sulphate must be used until the purpose is effected—that of thoroughly dilating the pupil. It must be remembered that a solution of this strength is quite poisonous, and care should be exercised in its employment. A solution of this strength should not be entrusted to the patient, and when dropped into the eye by the surgeon with a dropper, the end of the finger should compress the Punctum at the inner canthus and force the superfluous drops out at the external canthus, to prevent any possibility of atropia poisoning.

When it produces an irritation of the conjunctiva, combine with it boracic acid or make an ointment of it with vaseline. If it must be abandoned, we have other mydriatics, such as duboisia, hyoscyamus and gelseminum, that may be substituted.

It is, above all things, important to prevent adhesions between the iris and capsule, and this is very much less likely to occur—in fact, it is almost impossible to occur—when the pupil is well dilated.

In the first place, when the pupil is dilated the iris is removed very much further from the capsule, making adhesions more unlikely; and, in the second place, the area to be filled with inflammatory exudation is largely increased and the danger of occlusion of the pupil is correspondingly reduced.

Besides the above objects accomplished by the instillation of atropia, it is an anodyne and acts as a calmative to the pain, which is very severe in iritis. Atropia is to the eye what morphia is to the general system.

By contracting the iris the inflammation is directly attacked: because, the iris tissue being pressed upon, holds less blood and, of course, less exudation is the consequence.

All other treatment is secondary to this, with, perhaps, the exception of syphilitic iritis, where constitutional treatment is as important as the local. If, gentlemen, the case of iritis you are called upon to treat be in a sthenic person, you can add to the above treatment local blood-letting, either with leeches or, more preferably, the Henrtelup (an artificial leech).

If there be much pain, give opium in some of its forms, and do not be governed by the scales in making the dose, but by the effect. Of course, I do not mean by this that you shall not be very careful in administering opium or morphine in maximum doses, but a patient suffering from a severe iritis (especially the rheumatic form) can stand large doses of opiates—indeed, they are necessary—but watch the effects closely where you have given a maximum dose of any drug. The pain in iritis is more severe at night; hence, opiates are called for then.

The blinds should be drawn, or the shutters closed, not to darken the room, but to render the light soft. If the photophobia be so severe that no light can be borne, then bandage the eyes and exclude the light entirely, but do not make absolute darkness in the room and thus deprive the other portions of the body and the attendants of all light.

Warm applications are soothing, and this is best accomplished by laying over the eye a bag of hops the size of the hand, previously dipped into hot water and the superfluous water squeezed out.

You will often find that in traumatic and sthenic cases cold applications will prove more soothing. Always consult the feelings of the patient with reference to this point.

If you diagnose your case of iritis to be syphilitic, then begin at once on mercury and iodide of potash. It was formerly the custom to push the mercury to ptyalyzation, but now some claim that they have been successful without any mercury. I think the proper course lies between these two extremes. But, on the other hand, contrary to the recommendation of some writers upon this subject, I *push iodide of potash.* In one case of syphilitic condyloma, which occurred in my practice last month, I saved the eye, as I believe, with one-quarter of a pound of iodide of potash given in one week. In short, I made the tolerance of the stomach the only limit. I would not advise you, however, to give quite so much in the beginning, as you will find that *iodism* will usually be brought about short of this—and, again, the stomach will not usually bear so much.

In rheumatic iritis, give large doses of salicylate of sodium—as much as forty grains every four hours.

There is a serous form which is very chronic in its nature and seldom very painful. This form is very tedious to treat, and we have to rely upon alteratives and small doses of mercury.

